# Alpha-Herpesvirus Infection Induces the Formation of Nuclear Actin Filaments 

**DOI:** 10.1371/journal.ppat.0020085

**Published:** 2006-08-18

**Authors:** Becket Feierbach, Silvia Piccinotti, Margaret Bisher, Winfried Denk, Lynn W Enquist

**Affiliations:** 1 Department of Molecular Biology, Princeton University, Princeton, New Jersey, United States of America; 2 Max Planck Institute for Medical Research, Heidelberg, Germany; Washington University School of Medicine, United States of America

## Abstract

Herpesviruses are large double-stranded DNA viruses that replicate in the nuclei of infected cells. Spatial control of viral replication and assembly in the host nucleus is achieved by the establishment of nuclear compartments that serve to concentrate viral and host factors. How these compartments are established and maintained remains poorly understood. Pseudorabies virus (PRV) is an alpha-herpesvirus often used to study herpesvirus invasion and spread in the nervous system. Here, we report that PRV and herpes simplex virus type 1 infection of neurons results in formation of actin filaments in the nucleus. Filamentous actin is not found in the nucleus of uninfected cells. Nuclear actin filaments appear physically associated with the viral capsids, as shown by serial block-face scanning electron micropscopy and confocal microscopy. Using a green fluorescent protein-tagged viral capsid protein (VP26), we show that nuclear actin filaments form prior to capsid assembly and are required for the efficient formation of viral capsid assembly sites. We find that actin polymerization dynamics (e.g., treadmilling) are not necessary for the formation of these sites. Green fluorescent protein-VP26 foci co-localize with the actin motor myosin V, suggesting that viral capsids travel along nuclear actin filaments using myosin-based directed transport. Viral transcription, but not viral DNA replication, is required for actin filament formation. The finding that infection, by either PRV or herpes simplex virus type 1, results in formation of nuclear actin filaments in neurons, and that PRV infection of an epithelial cell line results in a similar phenotype is evidence that F-actin plays a conserved role in herpesvirus assembly. Our results suggest a mechanism by which assembly domains are organized within infected cells and provide insight into how the viral infectious cycle and host actin cytoskeleton are integrated to promote the infection process.

## Synopsis

Regulation of subcellular organization and transport is essential for control of crucial biological processes. However, our knowledge often is hampered because these processes tend to be transient and difficult to study. Studies of how opportunistic microbes hijack cellular machinery have provided insights into various normal cell processes. For example, studies with intracellular microorganisms, such as Listeria monocytogenes, Shigella spp., Rickettsia spp., and vaccinia virus, have significantly increased our understanding of the dynamic nature of the actin cytoskeleton. However, much less is known about subcellular organization and transport of cargo in the nucleus. The authors have discovered that alpha-herpesvirus infection of neurons leads to the transient formation of actin filaments in the nucleus. These filaments do not fill the nucleus, but rather associate with newly formed viral capsids. The nuclear actin filaments were initially identified in peripheral nervous system tissue using a new imaging technology, serial section scanning electron microscopy pioneered by Winfried Denk (a co-author). Their results suggest that nuclear actin filaments form as part of a general stress response to infection, but then are co-opted, perhaps to direct capsid transport to sites of budding along the nuclear envelope. This work illuminates a less well understood part of the viral life cycle and sets the stage for future work investigating control of how cargo is organized and moved in the nucleus.

## Introduction

Herpesviruses are widespread animal pathogens, producing a variety of diseases of medical and economic impact, including mucocutaneous infections, infections of the central nervous system, and occasionally infections of visceral organs. Herpesviruses are large double-stranded DNA viruses that replicate and encapsidate their genomes inside the nuclei of infected cells. The virions have a complex structure consisting of four components: membrane envelope, tegument, capsid, and core [1,2]. The core consists of the double-stranded DNA-genome. During assembly, the genome is packaged into a pre-formed capsid within nuclei of infected cells. The capsid is surrounded by a protinaceous layer called the tegument, and the entire particle is enclosed by a host-derived lipid envelope containing many different viral membrane proteins. The capsid is assembled in the nucleus as an immature procapsid and undergoes cleavage-induced rearrangements to form a mature capsid filled with DNA. The coordination of herpesvirus capsid assembly and subsequent nuclear egress is currently the subject of intense study [3–21].

Viral replication, late gene expression, and capsid formation take place within distinct intranuclear structures called replication compartments, originally defined by the localization of herpes simplex virus (HSV) single-stranded DNA-binding protein ICP8 [3–5,22,23]. Precursors to these compartments are distinct structures called pre-replicative sites, which form adjacent to cellular nuclear matrix-associated ND10 sites [4,19,20,24]. Pre-replicative sites undergo intranuclear movements that result in the formation of replication compartments [5]. Subsequently, replication compartments undergo movements that result in their coalescence into larger replication compartments [5]. During replication compartment formation, cellular chromatin is marginated, the nuclear lamina is disrupted, and the nucleus enlarges [6,9,10,14]. Although such domains certainly act to concentrate viral and cellular factors required for viral replication and assembly, the formation and maintenance of such compartments during viral assembly remain poorly understood.

The alpha-herpesvirinae subfamily includes pseudorabies virus (PRV), varicella-zoster virus, and herpes simplex virus type 1 and type 2 (HSV-1 and HSV-2). PRV has an exceptionally broad host range and is often used to study alpha-herpesvirus invasion and spread in the nervous system. Using a combination of serial-section scanning electron microscopy [25], confocal microscopy, and transmission electron microscopy (TEM), we show that PRV and HSV-1 infections of peripheral neurons result in the formation of nuclear actin filaments. Using PRV expressing a green fluorescent protein (GFP)-tagged VP26 capsid protein, we demonstrate by confocal microscopy that nuclear actin filaments associate with viral capsids and form prior to capsid assembly. By using the actin-depolymerizing drug latrunculin A, we show that F-actin is required for the efficient assembly of capsid-rich foci in the nucleus. In contrast, treatment with the actin-stabilizing drug jasplakinolide increased the number and the size of individual capsid-rich foci. We have found that GFP-VP26 nuclear foci co-localize with the actin motor myosin V, suggesting that viral capsids travel along nuclear actin filaments using myosin-directed transport. We have also found that the early stage of viral infection, but not viral genome replication and late gene expression, is required for actin filament formation. Our results suggest a mechanism by which assembly domains can be organized within infected cells and provide insight into how viral gene expression and host actin cytoskeleton may be integrated to organize and promote the infection process.

## Results

### Filaments Associate with Viral Capsids in Neuronal Nuclei

**Figure 1:**
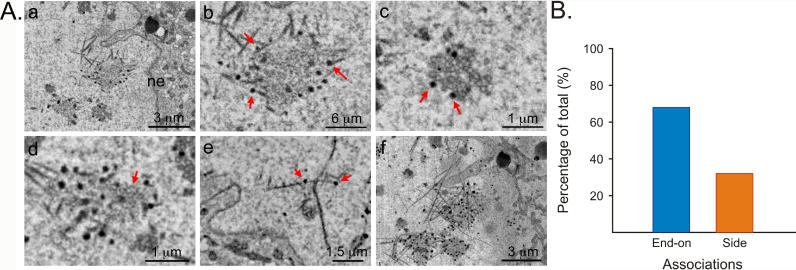
Filaments Associate with PRV Capsids in the Nuclei of Peripheral Neurons (A) SBFSEM images. Scale bar is indicated in each image. (a) Filaments (magnified in b) associated with genome-filled capsids in the nucleus. Note that the genome-packaged viral capsids appear as dark spots. Nuclear envelope is indicated (ne). Red arrows point out selected filament-capsid associations. (c) Enlargement of (a) showing aggregate of capsids, both empty and genome-filled. (d) Putative immature capsids (red arrow) appear associated with filamentous network. (e) Note side versus end-on associations between capsids and filaments. This image was processed by Volume Viewer (ImageJ plugin) in which a stack can be re-sliced along a selected plane. 30 consecutive images were rotated around the Z-axis and selected slice is shown. (f) A minimum-intensity projection from 30 consecutive layers in an image stack, taken 50-nm apart. (B) Histogram showing end-on versus side associations between nuclear filaments and viral capsids (*n* = 105).

To better understand PRV assembly in neurons, we used serial block-face scanning electron microscopy (SBFSEM) [25]. For SBFSEM, the sample is embedded in a block of resin and then imaged using back-scattered electrons, which shows the distribution of heavy atoms within a superficial layer of the block. A diamond knife blade is then driven across the surface of the sample block by an automated ultramicrotome drive to remove a thin (~50 nm) layer of resin. The cutting and imaging processes are iterated through a large number of cycles to obtain a well-registered volume image in the form of serial images, allowing the automated acquisition of 3-D datasets at nanoscopic resolution. We imaged mouse submandibular ganglia (SMG) infected with PRV. The SMG is a parasympathetic ganglion in the salivation circuit and has been used extensively to study synapse formation during development [26–28]. Using SBFSEM volume images, we noticed filaments in the nuclei of infected neurons that preferentially associated with genome-filled capsids, which appear as dark spheres (Figure 1A; see Figure 1Ac for enlargement of capsids; Video S1). Filaments were present only in the nuclei but not in the cytoplasm of infected cells (Video S2). In contrast, uninfected cells did not contain intranuclear filaments, as determined by analysis of serial SBFSEM images (compare Video S2 with Video S3). A stack of 65 images, sectioned at 50-nm thickness, clearly shows the aggregation of capsids, with genome-filled capsids at the edge of the aggregate, associating with fibers that extend to the nuclear envelope (Figure 1Af; Video S1). Most of the filament-capsid associations are end-on, although some capsids associated with the sides of filaments (Figure 1Ae and 1B). The filaments also associate with structures that appeared to be partially formed capsids (Figure 1Ad). A 2-D projection of the stack revealed a network of criss-crossing filaments surrounding accumulations of filled and unfilled capsids and other material (Figure 1Af). The filaments varied in length between 1 and 5 μm, with an average length of 3 μm (*n* = 30, SD ± 1.2 μm). The width of filaments ranged approximately from 25 to 100 nm (1–4 pixels), with the width varying along the filament length, suggesting the filaments are actually bundles composed of several filaments.

### Cultured Neurons Infected with PRV Have Numerous Polarized Nuclear F-Actin Filaments That Associate with Capsids

**Figure 2:**
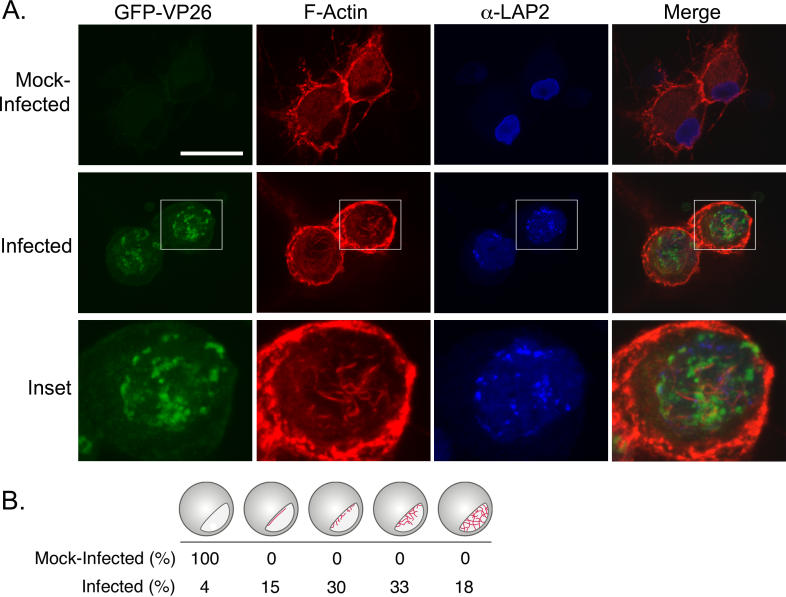
Actin Filaments Form in the Nuclei of PRV-Infected Neurons (A) Confocal images are 2-D projections from five consecutive layers in an image stack, taken 0.5 μm apart. GFP-VP26 is visualized by direct fluorescence. Scale bar = 20 μm. An enlargement of one of the nuclei is shown for clarity. Scale bar for enlargement = 10 μm. (B) Quantitation of actin filament formation within nuclei (infected versus mock-infected neurons).

In SBFSEM, the filaments were reminiscent of F-actin. To test the hypothesis that actin filaments form in the nuclei of PRV-infected cells, we fixed SMG and stained with AF568-phalloidin, which binds to F-actin, but not actin monomers. Unfortunately, immunofluorescence microscopy of whole ganglia resulted in high background at the surface and inefficient penetration of the phalloidin at the center of the ganglion (unpublished data). Consequently, we utilized in vitro dissociated cultures of superior cervical ganglion (SCG) neurons fixed and stained with AF568-phalloidin. The single-step growth kinetics of PRV in SCG neurons is similar to that of standard cell lines, with maximum production of infectious virus at 12 h post-infection (hpi) [29,30]. Confocal microscopy (versus wide-field microscopy) was required for the visualization of the nuclear actin filaments, due to the strong actin signal in the cytoplasm. In mock-infections, neurons showed normal actin staining with increased fluorescence in the actin-rich cortex (Figure 2, first row) and other structures, such as axons, known to be actin-rich. Mock-infected cells did not show any nuclear actin structures as shown by the antibody staining against a nuclear lamin-associated protein, α-LAP2 (Figure 2A, first row). We infected cells with a recombinant PRV strain expressing a GFP-VP26 (a capsid protein) fusion protein that is readily incorporated into infectious virions [31]. The number of GFP-VP26 proteins in each capsid is theoretically 900, enabling detection of individual capsids by fluorescence imaging [32]. The nuclear GFP-VP26 foci in Figure 2A are most likely aggregates of individual capsids. These foci may be analogous to HSV-1 VP26 foci, which serve as areas of capsid assembly [33]. Smaller punctae may be single whole, or partially formed capsids [31]. When infected cells were fixed at 15 hpi and stained with AF568-phalloidin, we saw actin filaments in the nucleus (Figure 2A, second and third rows). These additional filaments were restricted to the area within the α-LAP2 staining, indicating that the actin filaments were inside the nucleus. In PRV-infected neurons, 95% of nuclei contained actin filaments (*n* = 102 cells), with 51% of nuclei exhibiting extensive actin filament networks (Figure 2B). To verify that it was not the GFP-capsid fusion that triggered the formation of actin filaments, we infected cells with the wild-type PRV-Becker strain and found that these cells contained nuclear actin filaments to the same degree and number as the GFP strain (unpublished data).

**Figure 3:**
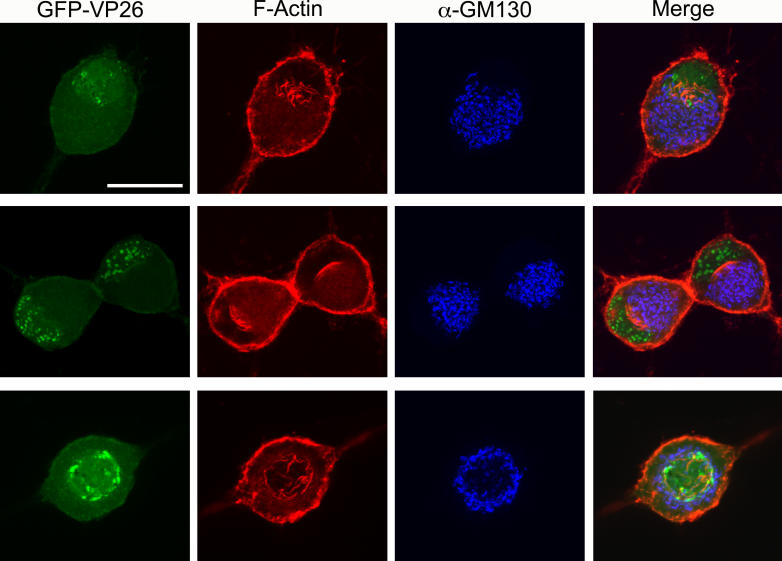
Polarity of Nuclear Actin Filaments Reflect the Overall Polarity of the Cell Neurons were stained with AF568-phalloidin, anti-GM130 to stain the Golgi. GFP-VP26 is visualized by direct fluorescence. Each image is a 2-D projection from four consecutive layers in a confocal image stack, taken 0.5 μm apart. Scale bar = 20 μm. Top two rows show polarized SCG neurons with one axon. Bottom row shows a single SCG neuron with two axons emanating from opposite sides of the cell body.

The vast majority of cells containing nuclear actin filaments (81%, *n* = 102 cells) showed a polarized distribution, with filaments primarily formed at one side of the nucleus (Figure 3). To determine whether this asymmetry reflects the overall polarity of the cell, we stained infected cells with an antibody to the Golgi marker, GM130. We found that the filaments were associated with the side of the nucleus that faced the Golgi apparatus (Figure 3, first and second row). However, in neurons with two axons on opposite sides of the cell body where the nucleus is located in the center of the cell body, the filaments appeared to emanate from all sides of the nuclear lamina; in these cells, the Golgi apparatus also formed a ring around the nucleus (Figure 3, bottom row). These findings suggest that the nuclear actin filaments reflect the overall polarity of the cell.

### Nuclear Actin Filaments Form before GFP-VP26 Foci and Co-Localize with GFP-VP26

**Figure 4:**
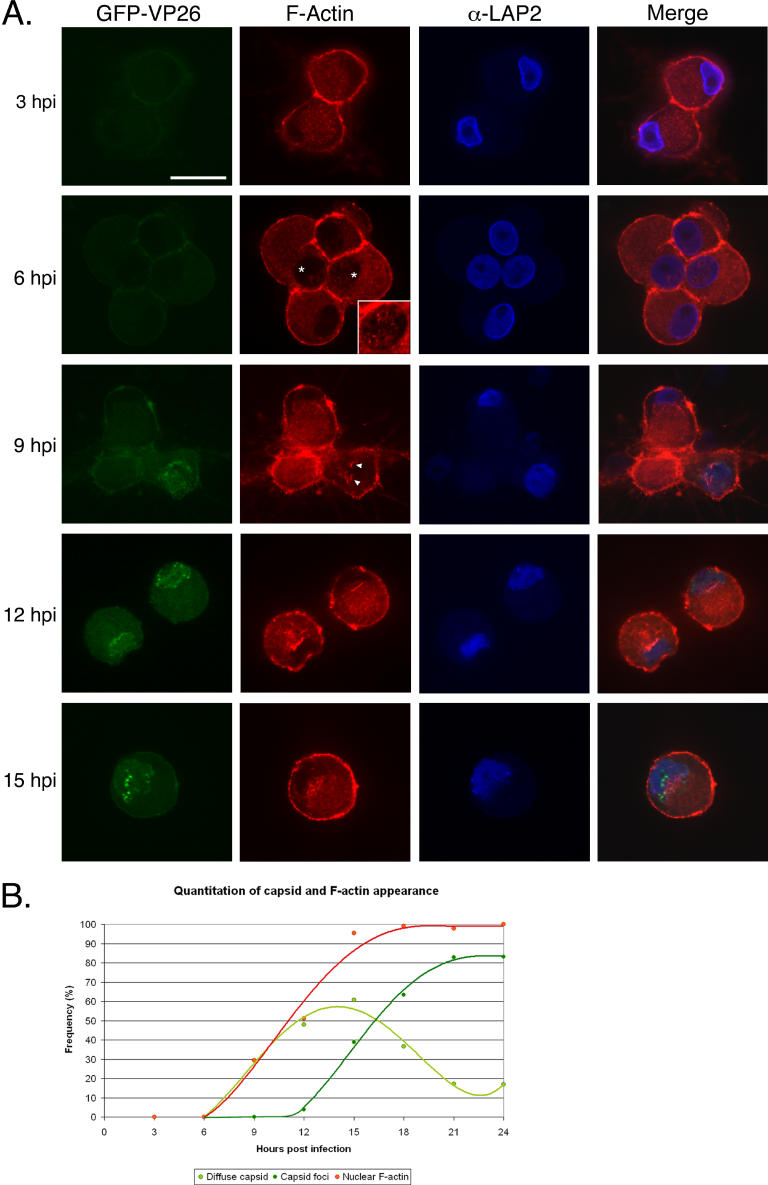
Time Course of Actin Filament Formation and Capsid Assembly in the Nucleus (A) SCG neurons were infected with PRV expressing GFP-VP26 at fixed times shown. The inset at 6 hpi is an enlargement of the nucleus from the cell on the right, which has small nuclear actin filaments. The brightness of the inset image has been enhanced in order to more clearly visualize the filaments. The arrowheads indicate actin filaments that appear to emanate from the nuclear envelope at 9 hpi. A single focal plane through the nucleus is shown. Scale bar = 20 μm. (B) Chart showing the relative formation of nuclear actin filaments, the presence of GFP-VP26 fluorescence, and emergence of GFP-VP26 foci over the course of infection.

To determine when actin filaments were formed during PRV infection, we recorded images of infected neurons every 3 h during a 24-h interval. At 3 hpi, we could not detect GFP-VP26 protein or actin filaments in the nucleus (Figure 4 and Table 1), suggesting either that actin filaments had not formed and GFP-VP26 had not yet been imported, or were present only in amounts too small to be detected by our methods. At 6 hpi, we still could not detect GFP-VP26 protein in the nucleus, but we did detect diffuse actin webs and very fine filaments in the nucleus (Figure 4 and Table 1). At 9 hpi, both GFP-VP26 protein and actin filaments were present in the same cells (29%, *n* = 99 cells) (Figure 4 and Table 1). By 12 hpi, 48% (*n* = 102) of the cells showed GFP-VP26 fluorescence, with 4% of cells having bright GFP-VP26 foci, and over 50% of the cells containing nuclear actin filaments (Figure 4 and Table 1). At 15 hpi, 95% of cells (*n* = 103) had nuclear actin filaments, with almost 40% of cells also containing GFP-VP26 foci (Figure 4 and Table 1). After 15 hpi, both GFP-VP26 foci and nuclear actin filaments became prominent, with the number of cells showing diffuse GFP-VP26 fluorescence decreasing and GFP-VP26 foci markedly increasing (Figure 4 and Table 1). Nuclear actin filaments appeared at the same time as diffuse GFP-VP26 fluorescence, but prior to the foci of GFP-VP26 (Figure 4B) suggesting that formation of GFP- VP26 into larger foci depends on F-actin. These foci may represent the accumulations of capsids in the nucleus that are seen by SBFSEM.

**Table 1:**
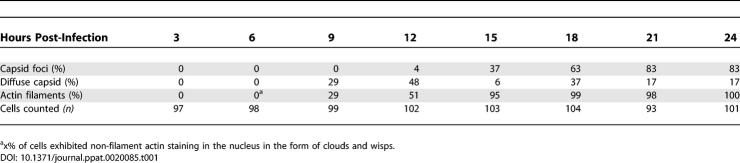
Quantitation of GFP-VP26 Foci and Nuclear Actin Filaments during Infection

**Figure 5:**
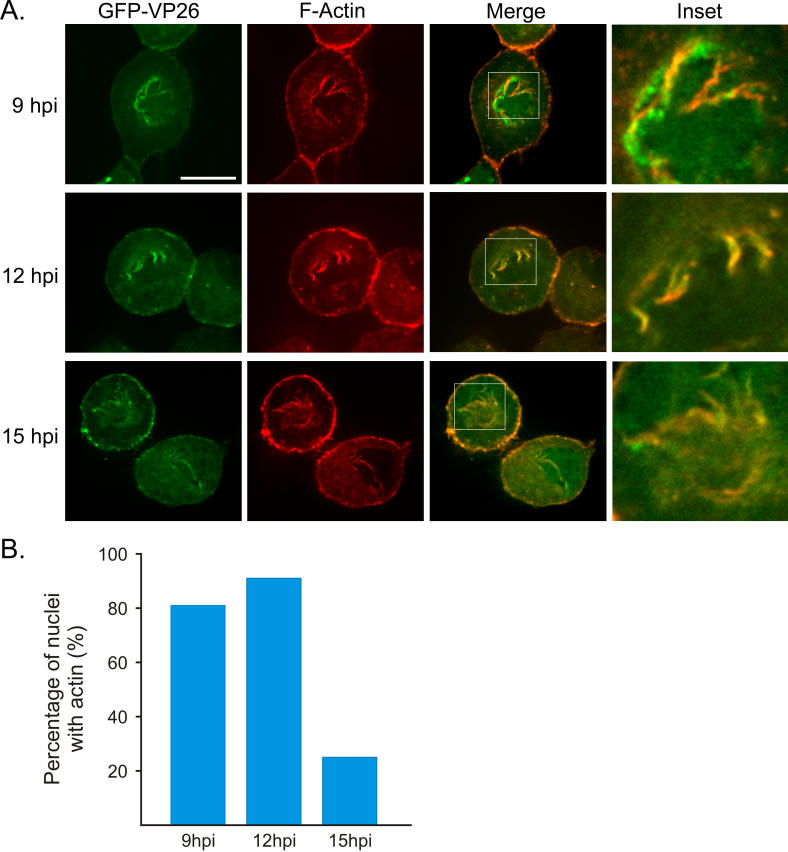
GFP-VP26 Co-Localizes with Nuclear Actin Filaments (A) Neurons were infected with PRV expressing GFP-VP26 and were fixed at time points shown. A single focal plane through the nucleus is shown, which can result in actin filaments appearing “discontinous” due to filaments weaving in and out of the plane of focus. An enlarged image of the nucleus (inset) is shown for clarity. Merged image was created in ImageJ and color adjusted linearly to appear yellow. Scale bar = 10 μm. (B) Histogram shows percentage of cells with co-localized GFP-VP26 and nuclear actin filaments in cells positive for nuclear actin at time points indicated. This histogram represents the percentage of cells within a population that show co-localization (this histogram does not show the degree to which GFP-VP26 and nuclear actin filaments are co-localized within a given cell).

During the 24-h time course following infection, we noticed precise co-localization between GFP-VP26 and actin filaments in the nucleus at specific time points: 9, 12, and 15 hpi (Figure 5). In some cells, the GFP-VP26 was discontinuous along the F-actin filament (Figure 5, 9 hpi); in other cells, the GFP-VP26 fluorescence was continuous along the entire filament length, with GFP-VP26 foci detected at the edges of the nuclear envelope (Figure 5, row 2). Some cells showed GFP-VP26 fluorescence directly adjacent to as well as aligned with the filaments. At 9 hpi, nuclear actin filaments co-localized with VP26 in 81% of neurons (*n* = 48 cells). The peak of co-localization occurred at 12 hpi, with 91% of neurons (*n* = 55 cells) containing nuclear actin filaments showing co-localization with VP26. At 15 hpi, the amount of co-localization had markedly decreased with now only 25% of neurons (*n* = 51 cells) with nuclear actin filaments showing co-localization. These data are in agreement with the recent finding that HSV-1 capsids undergo active directed movements that are sensitive to an actin depolymerizing drug, latrunculin A (latA) and most of which are movements away from GFP-VP26 foci and toward the nuclear periphery [34].

**Figure 9:**
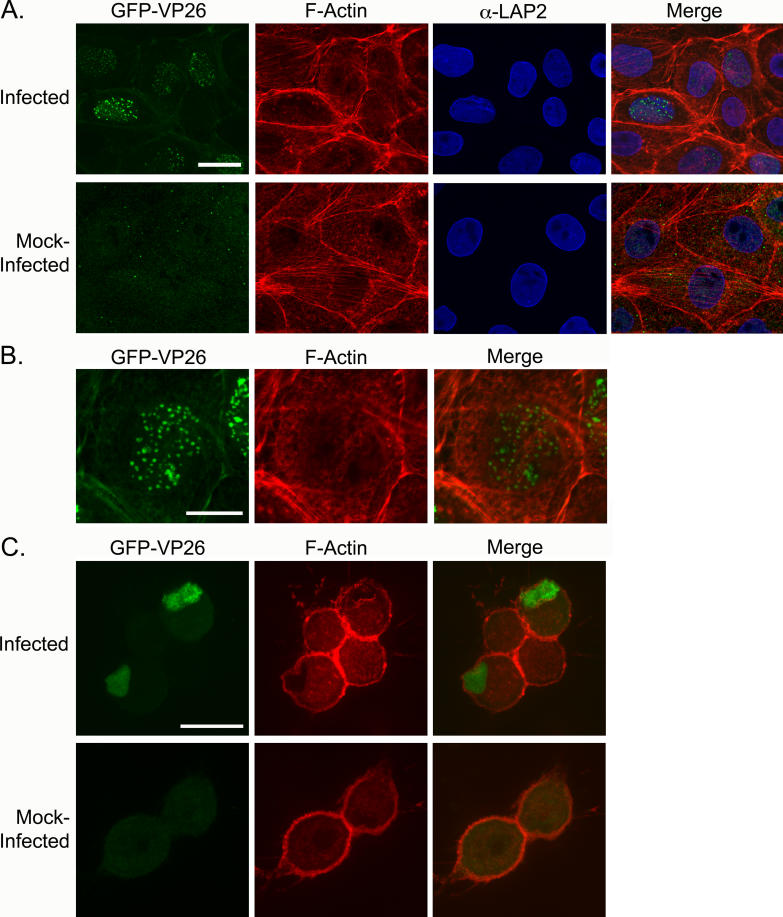
Conservation of Formation of Nuclear Actin Filaments (A) PK15s infected with PRV expressing GFP-VP26, fixed at 9 hpi. Asterisks show cells that are infected and show short actin filaments that appear to associate with nuclear membrane. Each image is a 2-D projection from four consecutive layers in an image stack, taken 0.1 μm apart. Scale bar = 20 μm. (B) Enlarged image of nucleus labeled with yellow asterisk in (A) Arrowhead indicates nuclear actin filaments. Scale bar = 10 μm. (C) SCG neurons infected with HSV-1 (KOS), fixed at 15 hpi. Cells were stained for the presence of viral capsid with anti-VP5 (capsid protein) antibody. Arrowheads indicate nuclear actin filaments. Each image is a 2-D projection from four consecutive layers in an image stack, taken 0.5 μm apart. Scale bar = 20 μm.

### Nuclear Actin Filaments Coordinate the Assembly of GFP-VP26 Foci

**Figure 6:**
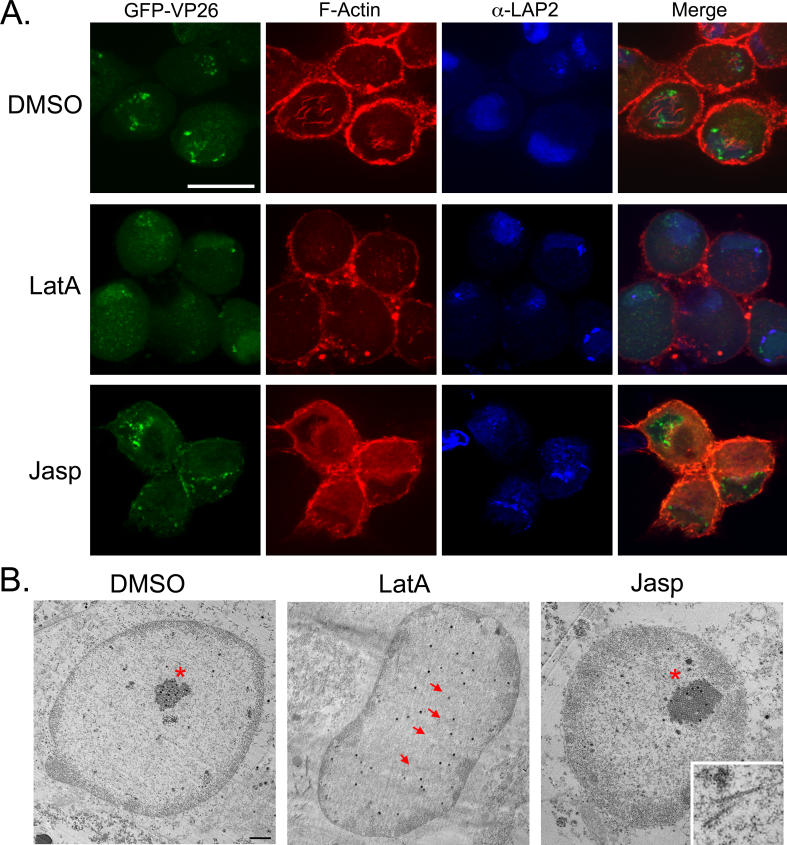
Drug Effects on Actin Filament Formation and Capsid Assembly Organization in the Nucleus Infected cells were treated with latA, jasp, or DMSO as indicated. (A) Each image is a 2-D projection from four consecutive layers in an image stack, taken 0.5 μm apart. Scale bar = 20 μm. The contrast of the α-LAP2 signal has been enhanced in jasp-treated cells. (B) TEM of infected cells treated with with latA, jasp, or DMSO as indicated. Asterisks indicate capsid assemblies; arrows point out individual capsids. Inset shows filaments from a different jasp-treated cell; arrows point to filaments. Scale bar = 2 μm.

To test whether nuclear actin filaments organize capsids in the nucleus, we treated cells with the actin-depolymerizing compound latA. We infected cells with PRV expressing GFP-VP26 and added 5 μm of latA at 3 hpi, after viral entry was complete. At 15 hpi, cells were fixed and then stained with AF-568 phalloidin. All nuclear actin filaments had depolymerized, yet some cortical and axonal actin structures remained intact (Figure 6, row 2). Cells treated with latA contained half as many nuclei with capsid foci (18% of latA-treated cells, *n* = 110 cells; 39% of untreated neurons at 15 hpi, *n* = 302; Table 1). At 15 hpi, 80% of latA-treated cells (*n* = 110 cells) exhibited only diffuse GFP-VP26 fluorescence throughout the nucleus, rather than discrete VP26 foci, compared with 61% of untreated neurons (*n* = 302). When we replaced the media containing latA at 15 hpi with fresh neuronal media and allowed the infection to progress until 24 hpi, we found that nuclear actin filaments and GFP-VP26 foci were restored to control levels (unpublished data). These results indicate that the establishment and/or maintenance of nuclear GFP-VP26 foci is partially dependent upon nuclear F-actin filaments.

To test whether F-actin polymerization dynamics are required for proper VP26 organization, we treated cells with the actin filament-stabilizing drug jasplakinolide (jasp). Addition of jasp prevents the remodeling of actin filaments and inhibits the intracellular movement of organisms (e.g., Listeria monocytogenes) that use actin polymerization. We infected cells with PRV expressing GFP-VP26 and added 100 nM of jasp at 3 hpi. At 15 hpi, we fixed the cells and stained them with AF-568 phalloidin. Since jasp binds the same domain on the actin filament as phalloidin, the intensity of phalloidin staining was markedly reduced, even though pre-existing actin structures are not affected by the drug [35]. When neurons were treated with jasp, there was an increase in neurons showing GFP-capsid foci (57% of jasp-treated neurons had foci, *n* = 100 cells; 39% untreated neurons at 15 hpi, *n* = 302) with a concomitant decrease in diffuse GFP-capsid fluorescence (43% of jasp-treated neurons showed diffuse GFP-capsid, *n* = 100 cells; 61% of untreated neurons at 15 hpi, *n* = 302; Table 1 and Figure 6). This result indicates that actin polymerization-based movement is not required for the establishment of GFP-capsid foci, and rather suggests that stabilizing actin filaments may actually promote or stabilize the assembly of GFP-capsid foci.

From the confocal microscopy results, we were unable to determine if latA treatment caused the disassembly of GFP-VP26-capsid foci or of the capsids themselves. To distinguish these possibilities, we examined infected latA-treated neurons by TEM and found that infected cells treated with latA had dispersed, but distinct capsids, rather than capsid accumulations, as seen in the DMSO control (Figure 6B; arrows highlight dispersed capsids; asterisk indicates capsid accumulation). That individual capsids were intact indicates that latA does not disrupt capsid structure. When infected neurons were treated with jasp, capsid accumulations were still present and were, in fact, larger than those in the DMSO control (Figure 6B, asterisk indicates capsid accumulation). In addition, we detected filaments in the jasp-treated cells that were similar in structure to those seen with SBFSEM (Figure 6B, inset). It is likely that filaments in the jasp-treated cells are due to its actin-stabilizing activity [35]. These data are in agreement with the confocal microscopy results indicating that actin filaments are involved in establishing and maintaining capsid foci.

### Myosin V Co-Localizes with GFP-VP26 Foci in the Nucleus

**Figure 7:**
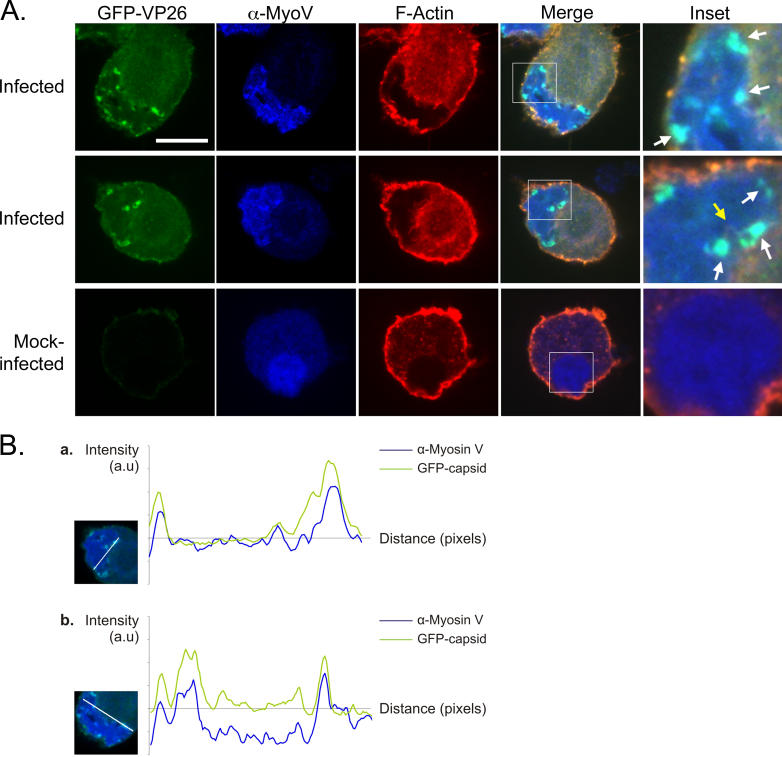
GFP-VP26 Co-Localizes with the Actin Motor Myosin V (A) Anti-myosin V antibody was used to stain for presence of myosin V. A single focal plane through the nucleus is shown. White arrows highlight areas of co-localization between myosin V and GFP-VP26 foci. Yellow arrow indicates nuclear actin filament associating with both myosin V and GFP-VP26 foci. Scale bar = 10 μm. (B) Profile plots of the GFP-VP26 (green) and anti-myosin V (blue) signal intensities along a straight line intersecting GFP-VP26 foci through the nucleus. Inset shows the trajectory of the straight line on a merged image of the nucleus. Signal intensity profile plots were obtained using ImageJ and corrected for background noise by subtracting the average intensity of the approximate nuclear area from the profile data. Curve points below zero correspond approximately with points outside the nucleus. Correlation coefficients between GFP-VP26 and myosin V for plots shown: (a) 0.87 and (b) 0.72.

The effects seen with latA and jasp provide support for a mechanism in which individual capsids are using nuclear actin filaments as tracks for directed transport. To understand the basis for these capsid movements, we sought to find a myosin motor that associates with the capsids. The three main families of myosin responsible for intracellular movements are myosin I, II (non-muscle), and V. Since a nuclear myosin I (NM1) has been implicated in chromatin rearrangements and interaction with actin in the nucleus, we stained for NM1 in the nucleus of infected neurons. We did not, however, find any co-localization between capsids and NM1 (unpublished data). We also did not detect any significant co-localization between capsids and myosin II (Figure S1). However, when we stained with antibodies against myosin Va, we detected significant co-localization with GFP-capsids, with the highest concentration of myosin Va at GFP-VP26 foci (Figure 7A). When we compared the localization of GFP-VP26 and myosin Va in merged images, the correlation coefficients were very high, indicating a high degree of signal overlap (0.87, 1.00 = 100% correlation; Figure 7B). In contrast, the correlation between GFP-VP26 and myosin II was very low (−0.08; Figure S1). A recent paper imaging intra-nuclear HSV-1 capsids showed capsid movements to be sensitive to BDM, a putative myosin ATPase inhibitor [34]. Our findings support this data and suggest that the capsids are moving along actin filaments using the actin-based motor myosin Va.

### Nuclear Actin Filament Formation Does Not Require Viral DNA Synthesis, but Does Require Protein Synthesis

**Figure 8:**
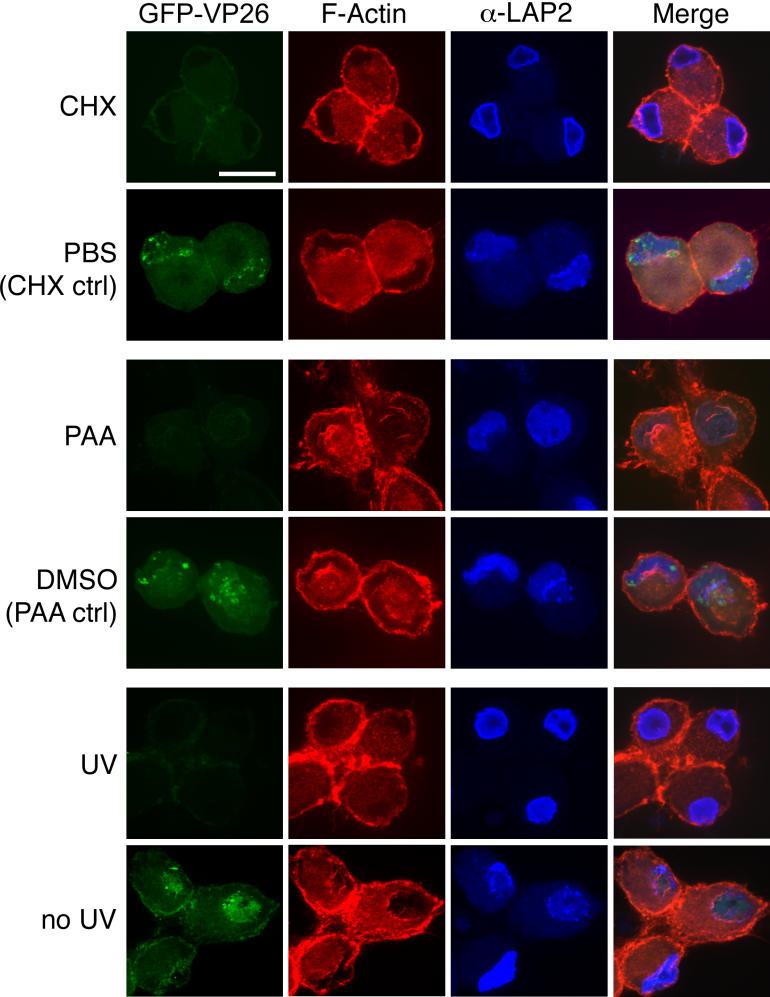
Host and Viral Requirements for Nuclear Actin Filament Formation Cells were treated with cycloheximide or PAA, as indicated. Alternatively, viral stocks were treated with UV irradiation. Each image is a 2-D projection from four consecutive layers in an image stack, taken 0.5 μm apart. Scale bar = 20 μm.

The following experiments address the viral and cellular requirements for actin filament formation. To test whether protein synthesis is needed for actin filament formation, we treated PRV-infected neurons with cycloheximide. Following 1 h pre-treatment with 100 μg/ml cycloheximide, neurons were infected with PRV expressing GFP-VP26 in the presence of cycloheximide. At 15 hpi, we fixed the cells and stained them with AF568-phalloidin. In cycloheximide-treated cells we did not detect any nuclear actin filaments (Figure 8), which shows that new protein synthesis is needed for the filament formation.

To test whether viral DNA synthesis is required for the formation of nuclear actin filaments, we treated cells with phosphonoacetic acid (PAA), a specific inhibitor of viral DNA synthesis [36,37]. We infected cells with PRV expressing GFP-VP26 and added 400 μM of PAA at 1 hpi. At 15 hpi, we fixed cells and stained them with AF568-phalloidin. Despite the inhibition of viral DNA synthesis, actin filaments were present at a frequency similar to DMSO-treated cells (86% of PAA-treated cells, *n* = 106; 95% of DMSO-treated cells, *n* = 91). We conclude that viral DNA synthesis is not required for formation of the actin filaments (Figure 8).

PAA treatment of infected cells does not prevent expression of immediate-early and early viral genes. To determine whether these viral gene products are required for actin filament formation, we inactivated PRV stocks with ultraviolet light (UV), which reduced infectivity by four orders of magnitude. UV-treated virions can still bind to and enter cells, but viral transcription and DNA replication are blocked. UV-treated virions also deliver tegument proteins after entry [38]. We did not detect nuclear actin filaments after infection by UV-treated virions (Figure 8), which indicates that new synthesis of immediate-early and/or early viral proteins is required for actin filament formation. Taken together, these results are evidence that viral and/or host protein synthesis is required for the formation of actin filaments.

### Nuclear Actin Filament Formation Is Not Specific for PRV or for Neurons

To test whether the formation of nuclear actin filaments is specific to neurons, we infected PK15 cells, a transformed porcine kidney epithelial cell line, with PRV and stained cells with AF568-phalloidin. Infected, but not uninfected, PK15 cells exhibited nuclear actin filaments (Figure 9A and 9B). But, in distinction to infected neurons, the nuclear filaments were finer and less organized in PK15 cells (Figure 9A and 9B). Furthermore, nuclear actin filament formation is not specific to PRV infections since we also observed the formation of nuclear actin filaments when we infected SCG neurons with HSV-1 (KOS) (Figure 9C). This suggests that nuclear actin filament formation is a mechanism that is conserved within the alpha-herpesvirus subfamily.

## Discussion

In this report, we show that herpesvirus infection induces the formation of actin filaments in the nucleus. The timing of filament formation (Figure 4), the association of capsids with filaments (Figures 1 and 5) and myosin V (Figure 7), and the dependence of VP26 organization on filaments (Figure 6), suggests that F-actin plays a key role in the formation and organization of viral assembly centers in the nucleus. Our results support the recent finding showing that directed movement of HSV-1 capsids in the nucleus is actin-, and likely, myosin-dependent [34]. We propose that nuclear actin filaments provide tracks for myosin—rather than actin polymerization-based movement, because elimination of actin dynamics by jasp did not disrupt VP26 localization (Figure 6). A diverse group of intracellular microorganisms, including Listeria monocytogenes, Shigella spp., Rickettsia spp., and vaccinia virus, utilize the host actin cytoskeleton to move within and spread between mammalian host cells. We believe that herpesviruses provide another distinct example of a pathogen appropriating the host actin cytoskeleton.

We first observed nuclear actin filaments in infected mouse SMG tissue in volume electron-microscopic images obtained using SBFSEM (Figure 1). In those images, we detected a network of filaments in the nucleus surrounding the PRV capsids present only in infected cells. Most of the filaments associated with individual genome-filled capsids, showing both end-on and side-on associations. Given the association of filaments exclusively with genome-filled capsids, we propose that the filaments play a role in capsid assembly and/or the transport of capsids in preparation for egress. Similar filaments described as “interwoven fine fibrils” have been found to associate with HSV-1 and HSV-2 nucleocapsids in infected CNS, PNS, and glial cell nuclei [39]. Thus, the formation of such filaments may be a conserved feature in the alpha-herpesvirus life cycle.

We tested the hypothesis that infection induces filament formation using dissociated, cultured peripheral SCG neurons. Neurons infected with PRV or HSV-1 contained a network of nuclear actin filaments, visualized by fluorescently labeled phalloidin (Figures 2 and 9). However, the extent to which actin filaments were present in the nucleus was variable: some cells contained a single large bundle running along one face of the inner nuclear membrane whereas others possessed larger, more complex structures. We do not yet understand the factors responsible for these differences, but amount of viral production or the size of the actin monomer pool could play a role. The average length of nuclear actin filaments seen by confocal microscopy (4.5 ± 1.9 μm) was similar to that seen with SBFSEM (3.0 ± 1.2 μm) and in both cases filaments specifically associated with capsids and were present only in infected cells. Thus, they may be the same structures. In PK15 cells, nuclear actin filaments appeared finer and less organized than those in SCG neurons, perhaps reflecting differences between polarized and non-polarized cells or primary and transformed cells.

Actin filaments associate with the nuclear lamina that faces the Golgi (Figure 3) which implies that, if actin filaments are indeed utilized for nuclear egress, capsids traveling along the filaments towards the nuclear membrane would emerge from the nuclear envelope ready to engage the secretory pathway. Consistent with this speculation, live imaging of HSV-1 capsids in the nucleus showed directed movements towards the nuclear envelope [34]. This result also suggests that if actin nucleators are involved in the formation of these filaments, then one would expect to find them asymmetrically localized on the nuclear lamina.

Since actin filaments are required for capsid foci formation, GFP-VP26 foci presumably reflect viral assembly sites as they have been observed and characterized in both PRV and HSV-1-infected cells [31,33]. Experiments with latA (Figure 6) suggest that actin filaments are important for establishment and/or maintenance of GFP-VP26 foci. A recent paper has shown that nuclear expansion in epithelial cells infected with HSV-1 is dependent on actin [10]. LatA also inhibits replication, compartment maturation, and chromatin dispersal [10], providing support for the notion that actin filaments provide a scaffold for viral assembly. Testing whether such a scaffold is also used for viral DNA replication will require further study.

Jasp, an inhibitor of actin treadmilling dynamics that stabilizes actin filaments, increased the number of cells containing GFP-VP26 foci by 30% (Figure 6). This finding suggests that capsid foci may be stabilized as a result of filament stabilization. At present, we do not know the degree to which the filaments are dynamic or if they are undergoing rapid turnover; Fluorescence Recovery after Photobleaching (FRAP) experiments with neurons expressing GFP-actin should answer this question. The effects of jasp imply that individual capsids use the actin filaments as tracks for directed movements rather than by using actin polymerization-based movement, which is distinct from the way that *Listeria* and vaccinia virus use the actin cytoskeleton. The result that capsids co-localize with the actin motor myosin Va (Figure 7) supports this idea. Class V myosins are among the most thoroughly studied forms of unconventional myosins and considerable evidence supports a role in transport of organelles and vesicles [40]. Myosin Va is a two-headed myosin that shows processive movement along actin filaments, similar to that of two-headed kinesins and dynein along microtubules [40,41]. Myosin Va is one of the fastest myosins, moving along actin filaments at a speed of 300–400 nm/s [42], which is comparable with the speed reported for directed movements of HSV-1 capsids [34].

We do not know whether actin filaments form as a result of rearrangement of the available nuclear monomer pool or if monomeric actin is recruited from the cytoplasm. Several recent publications [43–45] have demonstrated that actin is present in the nucleus and is critical for transcription, chromatin remodeling, mRNA export, and nuclear structure and integrity. However, the actin present in the nucleus may not be filamentous, since it is not recognized by phalloidin, which binds only to filaments more than seven monomers long [46]. By analogy, actin may also play a role in transcription of viral genes. β-actin has been shown to co-purify with RNA polymerase (I, II, and III), is a component of pre-initiation complexes, and appears to be recruited to promoters of genes about to be transcribed [47–49]. However, recent work [10] demonstrates that treatment with inhibitors of actin polymerization does *not* affect HSV-1 viral replication (cytochalasin D treatment even increases viral titer about 15 times). These observations taken together with our findings that actin filaments still form when viral DNA replication is prevented (Figure 8) suggest that actin plays an ancillary rather than an essential role in the virus life cycle.

Recent work also shows that host-derived actin is incorporated into the PRV virion and becomes an integral part of the outer tegument layer [50,51]. The amount of actin incorporated increased in the absence of VP22, one of the major tegument proteins, providing support for the view of the outer tegument layer as dynamic outer shell [50,51]. Virion-associated actin has been reported in other herpesviruses [1,52–56] and other enveloped viruses including paramyxovirus, retrovirus, and rhabdovirus [57–61]. Although actin incorporation may be required as a structural element of the virion, actin may also serve an additional function later in infection, such as nuclear egress or envelopment.

We do not know how actin filament formation occurs after infection, but we do know that new protein synthesis is required. We also know that at least one viral immediate-early or early protein is required (Figure 8). This protein may promote the formation of actin filaments directly, perhaps as an actin nucleator. Studies of the mammalian stress response have revealed that formation of nuclear actin filaments, increased nuclear invaginations, and decondensation of nucleoli, events all associated with viral infection, occur in response to heat shock [62]. During the heat-shock response and during HSV-1 infection, cellular chaperones Hsp70 and Hsp40 are redistributed to the nucleus during infection and co-localize with ICP0, adjacent to replication complexes, thereby promoting sequestration and compartmentalization of the nucleus [21]. Herpesvirus infection induces cellular stress responses, which may be exploited to concentrate viral and host proteins required for viral assembly and packaging. Consistent with this speculation, baculovirus-infected cells form nuclear actin filaments at the time of virus assembly; these filaments co-localize with nucleocapsids and the baculovirus major capsid protein has been shown to bind F-actin [63]. Whether actin filament formation is a viral-induced response or a general stress response, viruses that replicate in the nucleus may utilize these actin filaments as a scaffold for assembly and genome packaging. As a first step toward testing these ideas, it will be important to understand how viral capsids interact with nuclear actin filaments by identifying viral proteins that interact with actin and/or myosin.

## Materials and Methods

### Virus and cells.

The swine kidney epithelial cell line (PK15) was purchased from the American Type Culture Collection (CCL-22). All non-neuronal cells were cultured in Dulbecco's modified Eagle medium supplemented with 10% of fetal bovine serum and 1% penicillin/streptomycin. All PRV stocks were produced in the PK15 cell line. PRV stocks used in this report include PRV Becker, a virulent isolate [64] and PRV-GS443, a recombinant expressing GFP fused to the VP26 capsid protein [31].

### Infection of mouse SMG.

Each 6–8-wk-old C57 B6 mouse was anesthetized with 100 μl of a freshly prepared, sterile filtered solution of ketamine (100 ± 10 mg/kg)/xylazine (10 ± 1 mg/kg) by intraperitoneal (IP) injection. The neck of the mouse, from the base of the chin to just above the ribcage, was shaved using a platinum razor blade. The shaved area was prepared for surgery in the laminar flow hood using aseptic technique by applying disinfectant scrub and swabbing the area with 70% isopropyl alcohol. The mice were at a surgical plane of anesthesia prior to incising the neck region to expose the salivary glands. An approximately 1.5-cm incision was made with a sterile scalpel blade on a scalpel handle, with the skin grasped using forceps in order to ensure a shallow incision. The animal was monitored during the entire surgical procedure; if at any time the animal is no longer in a surgical plane of anesthesia (e.g., increased respiratory rate, movement), then we injected the animal with additional ketamine/xylazine or ketamine alone (up to 10–20 mg/kg) to induce a deeper plane of anesthesia. Four separate 2-l injections of PRV inoculum (diluted in sterile PBS) was injected into the submandibular glands. The incision was closed with 6–0 silk sutures. The mouse was administered an IP injection in the scapular region of 2.0 mg/kg of buprenorphine for prophylaxis against post-surgical pain. At 48 hpi, the mouse was euthanized by CO_2_ inhalation and fixed with 4% paraformaldehyde by cardiac perfusion. The salivary glands were surgically removed and the SMG dissected out on Sylgard plates. This experimental protocol related to animal use has been approved by the Institutional Animal Care and Use Committee of the Princeton University Research Board under protocol number 1539-AR1 in accordance with the regulations of the American Association for Accreditation of Laboratory Animal Care and those in the Animal Welfare Act (Public Law 99–198).

### Block-face serial section scanning electron microscopy.

SMG were stained in a manner similar to what is described for TEM. Post-staining, the infected ganglia were embedded in Epon resin (EM Sciences). The embedded samples were mounted on an aluminum rivet and trimmed following the procedure given in Denk and Horstman, 2004 [25]. All data shown were taken on an environmental SBFSEM with a field-emission electron gun (QuantaFEG 200, FEI, Eindhoven, The Netherlands) at a gas (H_2_0) pressure of 23 P, and an electron energy of 3.0 keV. The mounted, trimmed samples were placed on the SEM microtome and sequential images from the block-face were acquired in between cut cycles. The images were taken at a digital resolution of 26 nm/pixel. The data were analyzed using ImageJ 1.32j software (National Institutes of Health). The reslicing of image stacks (see Figure 1e) was done using the ImageJ Volume Viewer plugin, which interpolates the z-axis data so that the digital resolution matches that of the lateral direction.

### Neuron culture.

Detailed protocols for dissecting and culturing neurons are found in Ch'ng et al. [65]. Briefly, sympathetic neurons from the SCG were dissected from E15.5 to E16.5 pregnant Sprague-Dawley rats (Hilltop Labs Incorporated, Pennsylvania, United States) and incubated in 250 μg/ml of trypsin (Worthington Biochemicals, Lakewood, New Jersey, United States) for 10 min. 1 mg/ml of trypsin inhibitor (Sigma-Aldrich, St. Louis, Missouri, United States) was added to neutralize the trypsin for 3 min and then removed and replaced with neuron culture medium. Prior to plating, the ganglia were triturated into dissociated neurons using a fire-polished Pasteur pipette and then plated onto glass cover slips in a 35-mm plastic tissue culture dish coated with 500 μg/ml of poly-DL-ornithine (Sigma-Aldrich) diluted in borate buffer and 10 μg/ml of natural mouse laminin (Invitrogen, Carlsbad, California, United States). The neuron culture medium consists of Dulbecco's modified Eagle medium (Invitrogen) and Ham's F12 (Invitrogen) in a 1:1 ratio. The serum-free medium was supplemented with 10 mg/ml of bovine serum albumin (Sigma-Aldrich), 4.6 mg/ml glucose (J. T. Baker), 100 μg/ml of holotransferrin (Sigma-Aldrich), 16 μg/ml of putrescine (Sigma-Aldrich), 10 μg/ml of insulin (Sigma-Aldrich), 2 mM of L-glutamine (Invitrogen); 50 μg/ml or units of penicillin and streptomycin (Invitrogen), 30 nM of selenium (Sigma-Aldrich); 20 nM of progesterone (Sigma-Aldrich) and 100 ng/ml of nerve growth factor 2.5S (Invitrogen). After 2 d post-plating, the neuronal cultures are treated with 1 μM of an antimitotic drug called cytosine β-D-arabinofuranoside (Sigma-Aldrich) to eliminate any non-neuronal cells. The neuron culture medium was replaced every 3 d and cultures were kept in a humidified, CO2 regulated 37 °C incubator. This experimental protocol related to animal use has been approved by The Institutional Animal Care and Use Committee of the Princeton University Research Board under protocol number 1453-AR2 in accordance with the regulations of the American Association for Accreditation of Laboratory Animal Care and those in the Animal Welfare Act (Public Law 99–198).

### Viral infections.

Protocols for viral infection of neurons have been described by Ch'ng et al. [65]. All PRV infections of neuron cultures were carried out under high multiplicities of infection (MOI) unless otherwise stated. Briefly, neurons were cultured on glass cover slips in 35-mm dishes for approximately 2 wk prior to any experiment. The viral inoculum was diluted in 2% fetal bovine serum in Dulbecco's modified Eagle (GIBCO, San Diego, California, United States) and overlaid on the neuronal culture for 1 h in a humidified 37 °C incubator. After 1 h, the viral inoculum was removed and replaced with neuron medium. Infections usually lasted for 15 h (unless otherwise stated) before the samples were fixed and processed for staining and immunofluorescence. The production of infectious virus over time in SCGs has been characterized [30, 66].

### Antibodies and stains.

Antibodies used in this study include mouse monoclonal against lamin-associated protein 2 (LAP2) (BD Biosciences, Palo Alto, California, United States; used 1:500), anti-VP5 (major capsid protein) antibody and anti-GM130 antibody (BD Transduction laboratories; used 1:250). Myosin Va antibody against neuronal rat myosin V was generously provided by Paul Bridgman (Washington University, St. Louis, Missouri, United States) and used at 1:2000. Myosin II antibody against neuronal rat myosin II (Covance) was used at 1:500. Actin was detected by Alexa 568-phalloidin (Molecular Probes, Eugene, Oregon, United States) used at 1:40. All secondary Alexa fluorophores were purchased from Molecular Probes and used at 1:500 dilution.

### Fluorescence, immunofluorescence, and drug treatments.

All fluorescence experiments carried out were performed as follows. Dissociated neurons on glass cover slips were incubated in phosphate-buffered saline containing 3% bovine serum albumin and 0.5% triton for 10 min before the addition of primary antibodies for 1 h. After 1 h, the primary antibodies were removed and the sample was washed three times with phosphate-buffered saline containing 3% bovine serum albumin (and 0.5% saponin when noted in text). Next, secondary antibodies were added to the sample and incubated for 1 h. After 1 h, the secondary antibodies were removed and the sample was washed three times with phosphate-buffered saline containing 3% bovine serum albumin (and 0.5% saponin when noted in text). To stain for filamentous actin, Alexa-568-phalloidin was added to the cover slip at a concentration of 6.6 μg/ml. The cover slip was mounted on a glass slide using Aqua polymount (Polysciences, Warrington, Pennsylvania, United States) and allowed to dry for 24 h prior to imaging. To depolymerize actin, latrunculinA (latA; Molecular Probes) was dissolved in DMSO and added directly to the media at 3 hpi at a final concentration of 5 μM. Jasplakinolide (jasp; EMD Biosciences, Darmstadt, Germany) was dissolved in DMSO and was added directly to the media at 3 hpi at a final concentration of 100 nM. Phosphonoacetic acid (PAA; Sigma-Aldrich) was dissolved in DMSO and added directly to the media at 1 hpi at a final concentration of 400 μM. Cycloheximide was used at 100 μg/ml (10 mg/ml stock dissolved in PBS). Cells were pre-treated with cycloheximide in neuronal media for 1 h and then infected with viral inoculum containing cycloheximide. After 1 h, the viral inoculum was removed and replaced with neuron medium containing cycloheximide. For UV-inactivation, we exposed aliquots of PRV443L strain to UV light using a UV Stratalinker 1800 (Stratagene, La Jolla, California, United States). We used a dosage of UV that reduced the titer by approximately 1,000-fold.

Samples were imaged with a Perkin-Elmer (Wellesley, California, United States) RS3 spinning disk confocal microscope side-mounted on a TE200-S Nikon Eclipse microscope (Tokyo, Japan) with an Argon/Krypton laser producing excitation lines of 488, 568, and 647 nms. Optical sections were acquired in 0.1, 0.25, or 0.5 μm steps, as stated. 2-D projections of confocal stacks and channel merges were created by ImageJ 1.32j software (National Institutes of Health). All figures were assembled in Adobe Photoshop 7.0.1. Alterations to image brightness and contrast were conducted in a linear manner and were applied equally to controls, except where otherwise noted. Supplemental video was assembled in ImageJ and converted to QuickTime format.

### TEM.

Whole SCG were cultured for 2 wk on Aclar (EM Sciences) in a manner similar to what is described above for glass cover slips (except without dissociation). Neurons were infected at a high MOI and treated with latA as described above. After 15 hpi, the plates were washed twice with phosphate-buffered saline, fixed with 2% glutaraldehyde in 0.2 M sodium cacodylate buffer (pH 7.2) for 4 h, and post-fixed with 1% osmium tetroxide in sodium veronal buffer for 1 h on ice. Samples were then rinsed with sodium veronal buffer four times and incubated with 0.25% toluidine blue in 0.2 M cacodylate buffer (pH 7.2) for 1 h; the staining solution was then removed with four rinses of sodium veronal buffer (pH 7.2), followed by four rinses with 0.05 M sodium maleate buffer (pH 5.1). Overnight incubation with 2% uranyl acetate in 0.05 M sodium maleate buffer was done in the dark followed by four rinses with 0.05 M sodium maleate buffer (pH 5.1). The fixed samples were then dehydrated with ethyl alcohol, embedded in Epon resin (EM Sciences) and cut into 70-nm sections using a Reichert Ultracut E ultramicrotome.

## Supporting Information

Figure S1 Profile Plots of the GFP-VP26 (Green) and Anti-Myosin II (Blue) Signal Intensities along a Straight Line Intersecting GFP-VP26 Foci through the Nucleus Inset shows the trajectory of the straight line on a merged image of the nucleus. Signal intensity profile plots were obtained using ImageJ and corrected for background noise by subtracting the average intensity of the approximate nuclear area from the profile data. Curve points below zero correspond approximately with points outside the nucleus. Correlation coefficients between GFP-VP26 and myosin II for plots shown: (A) −0.11 and (B) −0.18.
(9.9 MB TIF)

Video S1 QuickTime Video of a SBFSEM Stack of 65 Serial Sections from an Infected Cell, Sectioned at 50 nm The volume shown is at the inner edge of the nucleus, with the nuclear envelope at the right-hand side of the image. This video is a cropped substack from Video S2.
(3.1 MB MOV)

Video S2 QuickTime Video of a SBFSEM Stack Comprising 100 Serial Sections from an Infected Cell, Sectioned at 50 nm Lower half of the cell was not obtained during image acquisition.
(944 KB MOV)

Video S3 QuickTime Video of a SBFSEM Stack Comprising 150 Serial Sections from an Uninfected Cell, Sectioned at 50 nm (1.4 MB MOV)

## Abbreviations

GFP: green fluorescent protein
hpi: hours post-infection
HSV-1: herpes simplex virus type 1
jasp: jasplakinolide
lat A: latrunculin A
PAA: phosphonoacetic acid
PRV: pseudorabies virus
SBFSEM: serial block-face scanning electron microscopy
SCG: superior cervical ganglion
SMG: submandibular ganglion
TEM: transmission electron microscopy
UV: ultraviolet light
