# Correction: Alpha-Herpesvirus Infection Induces the Formation of Nuclear Actin Filaments

**DOI:** 10.1371/journal.ppat.0020103

**Published:** 2006-09-29

**Authors:** Becket Feierbach, Silvia Piccinotti, Margaret Bisher, Winifred Denk, Lynn W Enquist

In *PLoS Pathogens*, volume 2, issue 8: DOI: 10.1371/journal.ppat.0020085

Figure 9 did not include the asterisks and arrowheads that were referred to in its legend. The original legend and correct version of Figure 9 are included below.

## Figures and Tables

**Figure 9:**
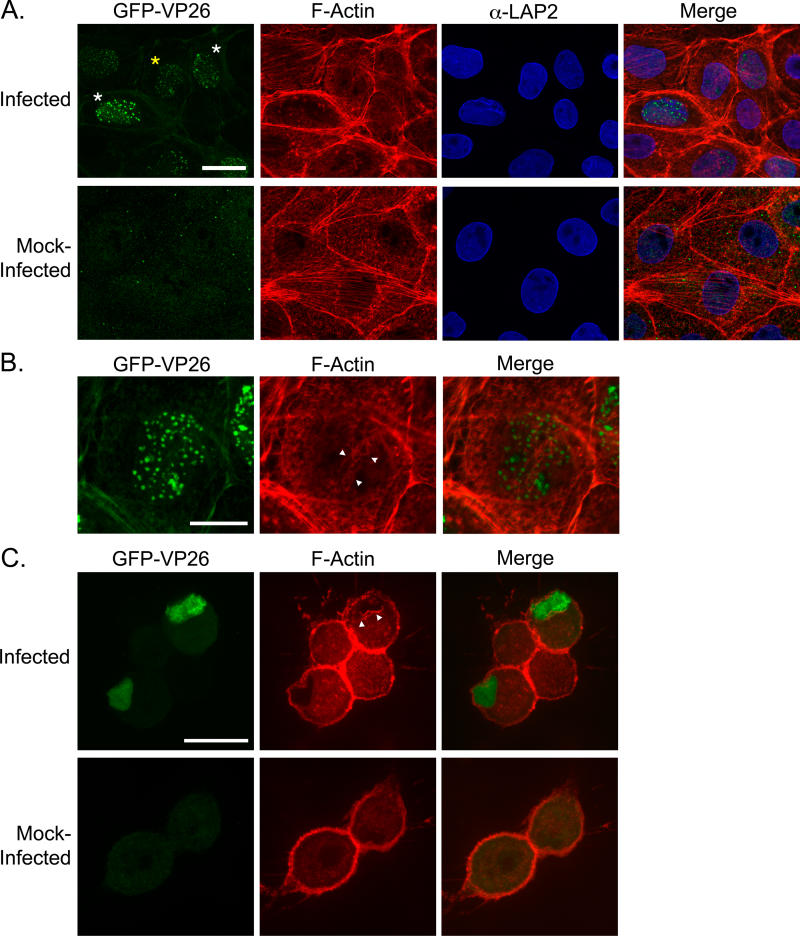
Conservation of Formation of Nuclear Actin Filaments (A) PK15s infected with PRV expressing GFP-VP26, fixed at 9 hpi. Asterisks show cells that are infected and show short actin filaments that appear to associate with nuclear membrane. Each image is a 2-D projection from four consecutive layers in an image stack, taken 0.1 μm apart. Scale bar = 20 μm. (B) Enlarged image of nucleus labeled with yellow asterisk in (A). Arrowhead indicates nuclear actin filaments. Scale bar = 10 μm. (C) SCG neurons infected with HSV-1 (KOS), fixed at 15 hpi. Cells were stained for the presence of viral capsid with anti-VP5 (capsid protein) antibody. Arrowheads indicate nuclear actin filaments. Each image is a 2-D projection from four consecutive layers in an image stack, taken 0.5 μm apart. Scale bar = 20 μm.

